# Tottenham Hospital

**Published:** 1904-11-12

**Authors:** 


### TOTTENHAM HOSPITAL.

### £4,000 FOR THE EXTENSION FUND.

A festival dinner in aid of the Tottenham Hospital Extension Fund was held at the Savoy Hotel on Wednesday, 2nd inst., Lord Burnham presiding, and resulted in a sum of £4,003 being collected towards this purpose. The hospital, which is being enlarged from 70 to 120 beds, is asking for £20,000 towards the enlargement, and of this sum the hospital has now raised £14,000, made up of £4,000 from the King's Fund, £5,500 from the Salters' Company, £500 from the Ecclesiastical Commissioners, and £4,000 the amount collected on Wednesday.

In proposing the loyal toasts, Lord Burnham, alluding to the charitable work of the King, said that the amount collected by the King's Fund since its establishment in 1897 was £1,100,000, of which it had distributed £500,000. All knew how heartily the Queen joined in the good work done by the King. The Prince of Wales had taken up the King's work in connection with the King's Fund, and he was assisted in turn by the Princess of Wales and the other members of the Royal Family. The name of one member of the Royal Family should be specially mentioned with gratitude in connection with that hospital, and that was the name of the gentle and courteous Princess Louise, Duches of Argyll, who is the president of the institution and took the very keenest interest in its welfare. (Applause.)

Subsequently Lord Burnham, in proposing "Prosperity to the Hospital," gave a brief history of the Tottenham Hospital which had had a career of uninterrupted success. It was auspicious in its origin, fortunate in its reorganisation, happy in the character of its site—which had been given by Mr. John Morley—happier still in the number of its benevolent friends, and happiest of all in the great and beneficent work it was enabled to do. (Applause.) It was a piece of good fortune that the year of its foundation was nearly, if not quite, the year when Lord Lister made known to the world his grand discovery of antiseptic, or, as it is now called, aseptic surgery. Its patients had had the benefit and advantage of two of the greatest discoveries in the history of medical science—Pasteur's germ theory and Lister's glorious doctrine which had almost robbed surgery of its terrors. (Hear, hear.) Its progress as an institution had been well defined. Though its beginning was small, its success, he believed, was immediate. First it was a home for waifs and strays, then it was a sisterhood of Protestant ladies who devoted their means and their lives to works of charity. Next was established a single ward in which these ladies practised the gentle art of nursing. Finally the institution was transferred to Tottenham and became a hospital proper. These ladies were really the pioneers of nursing reform, and their fame speedily became national. Wherever an epidemic broke out those who had to deal with it were keen and eager to secure the services of the Tottenham nurses. Their fame even became international—("hear, hear")—for when the war broke out in 1870 they were to be found upon the battlefields in France, and amongst their most cherished possessions was an autograph letter from the German Empress Augusta conveying to them the thanks of the Emperor for the services of these ladies in the field, and accompanied by a Cross of Merit. (Applause.) The constituency of the hospital consisted of Tottenham, Hornsey, Edmonton, Stoke Newington, Stamford Hill, and part of Walthamstow, districts which not so long ago were hamlets and villages, but had now grown to large towns with a collective population of half a million, to whom it was their duty to bring the highest medical skill and the largest surgical experience. Their district was as large as Edinburgh, Dublin, and Belfast, three times as large as Newcastle, and next in size to Glasgow, Manchester, and Birmingham.

Mr. John Langton and Sir Francis Cory-Wright, Bart., responded. The latter said that they had room for a hospital of 300 beds on their site, but at the present time it was proposed to enlarge the hospital to 120 beds. Plans and specifications for the enlargement were ready and tenders were coming in shortly.

Lord George Hamilton proposed and Sir Edmund Hay Currie responded to the toast of "The Visitors."

Other toasts followed.

